# Telomere-dependent and telomere-independent roles of RAP1 in regulating human stem cell homeostasis

**DOI:** 10.1007/s13238-019-0610-7

**Published:** 2019-02-22

**Authors:** Xing Zhang, Zunpeng Liu, Xiaoqian Liu, Si Wang, Yiyuan Zhang, Xiaojuan He, Shuhui Sun, Shuai Ma, Ng Shyh-Chang, Feng Liu, Qiang Wang, Xiaoqun Wang, Lin Liu, Weiqi Zhang, Moshi Song, Guang-Hui Liu, Jing Qu

**Affiliations:** 1 State Key Laboratory of Stem Cell and Reproductive Biology, Institute of Zoology, Chinese Academy of Sciences, Beijing, 100101 China; 2 Advanced Innovation Center for Human Brain Protection, National Clinical Research Center for Geriatric Disorders, Xuanwu Hospital Capital Medical University, Beijing, 100053 China; 3 National Laboratory of Biomacromolecules, CAS Center for Excellence in Biomacromolecules, Institute of Biophysics, Chinese Academy of Sciences, Beijing, 100101 China; 4 State Key Laboratory of Medicinal Chemical Biology, Nankai University, Tianjin, 300071 China; 5 State Key Laboratory of Membrane Biology, Institute of Zoology, Chinese Academy of Sciences, Beijing, 100101 China; 6 University of Chinese Academy of Sciences, Beijing, 100049 China; 7 Institute for Stem cell and Regeneration, Chinese Academy of Sciences, Beijing, 100101 China; 8 Key Laboratory of Regenerative Medicine of Ministry of Education, Institute of Aging and Regenerative Medicine, Jinan University, Guangzhou, 510632 China; 9 Beijing Institute for Brain Disorders, Capital Medical University, Beijing, 100069 China

**Keywords:** RAP1, stem cell, telomere, RELN, methylation

## Abstract

RAP1 is a well-known telomere-binding protein, but its functions in human stem cells have remained unclear. Here we generated RAP1-deficient human embryonic stem cells (hESCs) by using CRISPR/Cas9 technique and obtained RAP1-deficient human mesenchymal stem cells (hMSCs) and neural stem cells (hNSCs) via directed differentiation. In both hMSCs and hNSCs, RAP1 not only negatively regulated telomere length but also acted as a transcriptional regulator of *RELN* by tuning the methylation status of its gene promoter. RAP1 deficiency enhanced self-renewal and delayed senescence in hMSCs, but not in hNSCs, suggesting complicated lineage-specific effects of RAP1 in adult stem cells. Altogether, these results demonstrate for the first time that RAP1 plays both telomeric and nontelomeric roles in regulating human stem cell homeostasis.

**Electronic supplementary material:**

The online version of this article (10.1007/s13238-019-0610-7) contains supplementary material, which is available to authorized users.

## INTRODUCTION

Telomeres, the short repeat sequences at the chromosome ends in eukaryotic organisms (Greider, 1991; Blackburn, 2001), are indispensable for the maintenance of genome stability (Londono-Vallejo, 2004; Zhang et al., 2018). Telomere erosion induces cellular senescence in human cells (Proctor and Kirkwood, 2003; Lopez-Otin et al., 2013; Xu et al., 2013; Blackburn et al., 2015; Bourgeron et al., 2015; Zhu et al., 2018). Shelterin, also known as telosome, is a protein complex recruited by the telomeres. Shelterin is involved in the maintenance of advanced telomere structures and regulates the telomere niche via interaction with numerous protein components (Palm and de Lange, 2008; Xin et al., 2008; Bandaria et al., 2016). Human shelterin is composed of six components: RAP1, TRF1, TRF2, TIN2, TPP1 and POT1 (Schmutz and de Lange, 2016).

RAP1 (repressor/activator protein 1), also known as TERF2IP (TERF2 interaction protein), was first reported as a transcriptional regulator in *Saccharomyces cerevisiae* (Shore and Nasmyth, 1987). RAP1 is an evolutionarily conserved protein (Khurana et al., 2013; Kabir et al., 2014) that contains BRCT, Myb and C-terminal protein interaction domains (Kabir et al., 2010). RAP1 regulates telomeres by directly binding to double-stranded telomeric DNA (budding yeast) or interacting with a group of homologs consisting of Taz1 (fission yeast), TRF (trypanosome), TRFA (zebrafish) or TRF2 (mammals) (Kyrion et al., 1993; Kanoh and Ishikawa, 2001; Yang et al., 2009; Wagner et al., 2017). In yeast, RAP1 is implicated in the regulation of telomeric heterochromatin status by recruiting Sir2/3/4 protein complex (Moretti and Shore, 2001; Doerks et al., 2002); RAP1 deficiency leads to excessive telomere extension (Luo et al., 2002). However, the role of mammalian RAP1 is controversial. RAP1 deficiency results in shortened telomeres only in certain mouse tissues (Martinez et al., 2010, 2016). Similarly, in immortalized human cell lines, its deficiency causes telomere elongation in some cases, but exerts no effect on telomere length in other cases (Li and de Lange, 2003; O’Connor et al., 2004; Kabir et al., 2014; Kim et al., 2017). In addition to the role in regulating telomere length, RAP1 has also been reported to suppress the expression of telomeric repeat-containing RNA (TERRA) and subtelomeric genes (Nanavaty et al., 2017). Recently, emerging evidences have suggested that mammalian RAP1 may also play a nontelomeric role by occupying specific extratelomeric DNA regions as a transcriptional factor and regulating gene expression (Martinez et al., 2010, 2013, 2016; Yang et al., 2011). However, the underlying molecular mechanisms remain to be elucidated.

Senescence or exhaustion of adult stem cell pools is considered as a hallmark of aging (Liu et al., 2011, 2014; Lopez-Otin et al., 2013; Goodell and Rando, 2015; Zhang et al., 2015; Pan et al., 2016; Ren et al., 2017b; Yang et al., 2017; Wang et al., 2018b; Wu et al., 2018). In the search for therapeutic modalities to revitalize adult stem cells, telomere extension has attracted attention, but there was a lack of safe strategies and further validation. In this study, we found that RAP1 regulated human stem cell senescence in both telomere-dependent and telomere-independent manners. We knocked out RAP1 in hESCs by the CRISPR/Cas9 technique and differentiated RAP1-deficient hESCs into two different types of human adult stem cells, hMSCs and hNSCs. RAP1 deficiency was sufficient for telomere extension in both hMSCs and hNSCs, but delayed senescence only in hMSCs. We further identified that *RELN* was silenced with promoter hypermethylation in RAP1-deficient cells and that the RAP1-RELN pathway partially contributed to the regulation of senescence in hMSCs.

## RESULTS

### RAP1-deficient hESCs maintained pluripotency

**Figure 1:**
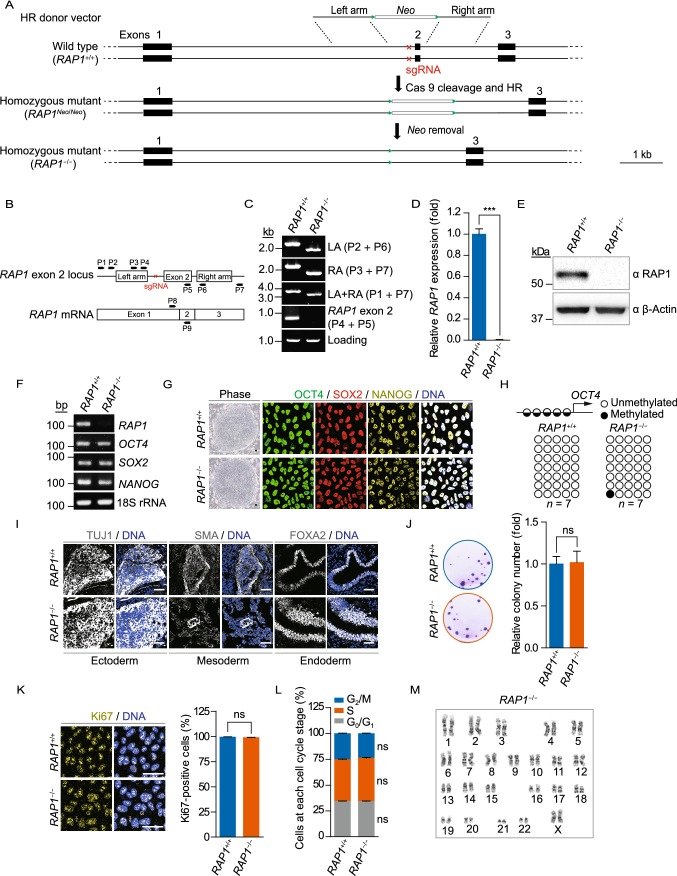
Generation and characterization of *RAP1*^−/−^ hESCs. (A) Schematic representation of the deletion of the exon 2 of *RAP1* in hESCs via CRISPR/Cas9-facilitated HR. The green triangles represented FRT sites and the red cross demonstrated the region of sgRNA. (B) Schematic representation of the primers used for genomic PCR and qRT-PCR to confirm *RAP1* knockout. (C) Genomic PCR analysis demonstrated that the exon 2 of *RAP1* was deleted from the genome. LA and RA represented left and right homology arm, respectively. (D) qRT-PCR analysis demonstrated the deletion of *RAP1* at the transcriptional level in *RAP1*^−/−^ hESCs by primers P8 and P9. Data were presented as the mean ± SEM, *n* = 3. ***P < 0.001. (E) Western blotting analysis verified the absence of RAP1 in *RAP1*^−/−^ hESCs. β-Actin was used as a loading control. (F) RT-PCR for pluripotency markers demonstrated comparable expression in WT and *RAP1*^−/−^ hESCs. 18S rRNA was used as a loading control. (G) Representative brightfield and immunofluorescence micrographs of WT and *RAP1*^−/−^ hESCs showed normal morphology of hESCs and comparable expression of pluripotency markers, respectively. Scale bar, 50 µm. (H) DNA methylation status of the *OCT4* promoter in WT and *RAP1*^−/−^ hESCs showed normal hypomethylation. (I) Immunostaining of representative markers of the three germ layers in teratomas formed by WT and *RAP1*^−/−^ hESCs. Scale bar, 50 µm. (J) Clonal expansion analysis of WT and *RAP1*^−/−^ hESCs. Data were presented as the mean ± SEM, *n* = 3. NS, not significant. (K) Immunostaining of the proliferation marker Ki67 in WT and *RAP1*^−/−^ hESCs. Scale bar, 50 µm. Data were presented as the mean ± SEM, *n* = 6. NS, not significant. (L) Cell cycle analysis of WT and *RAP1*^−/−^ hESCs. Data were presented as the mean ± SEM, *n* = 3. NS, not significant. (M) Karyotype analysis of *RAP1*^−/−^ hESCs showed normal karyotype. *n* = 10

To study the biological functions of human RAP1, we generated RAP1-knockout hESCs by deleting the exon 2 of *RAP1* (Kabir et al., 2014) via CRISPR/Cas9-facilitated homologous recombination (HR) (Wang et al., 2018a, b) (Fig. 1A). Biallelic deletion of the exon 2 of *RAP1* was confirmed by genomic PCR (Fig. 1B and 1C). Moreover, the successful ablation of RAP1 mRNA and protein was validated by quantitative reverse transcription PCR (qRT-PCR) and Western blotting (Fig. 1D and 1E).

*RAP1*^−/−^ hESCs exhibited normal colony morphology, expressed the pluripotency markers OCT4, SOX2 and NANOG (Fig. 1F and 1G) and maintained hypomethylation at the *OCT4* promoter (Fig. 1H). Meanwhile, teratoma analysis proved that *RAP1*^−/−^ hESCs were able to differentiate into endoderm, mesoderm and ectoderm lineages *in vivo* (Fig. 1I). Normal proliferation ability was verified via clonal expansion assay, Ki67 immunostaining, and cell cycle analysis (Fig. 1J–L). In addition, *RAP1*^−/−^ hESCs maintained normal karyotype (Fig. 1M). Taken together, these results indicate that *RAP1*^−/−^ hESCs maintained normal pluripotency and self-renewal capability.

### RAP1 deficiency delayed hMSC senescence

**Figure 2:**
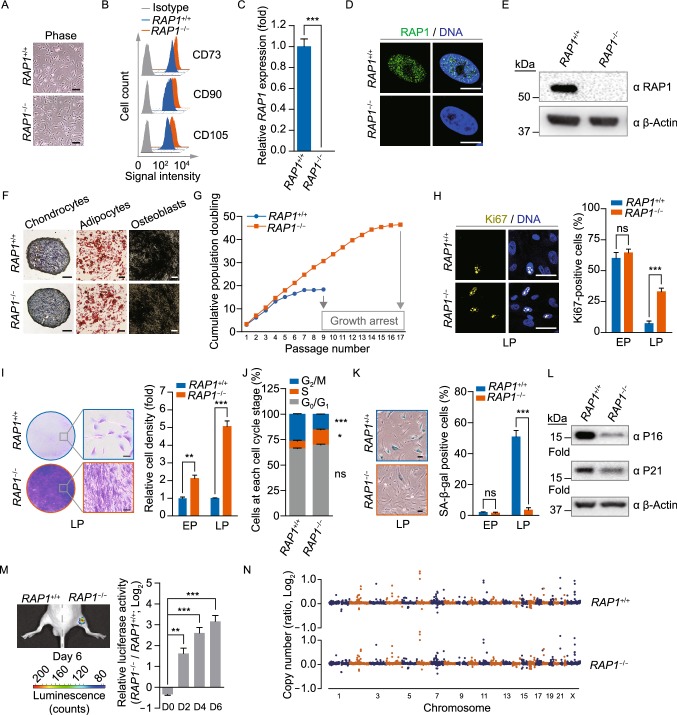
*RAP1*^−/−^ hMSCs exhibited retarded cellular senescence. (A) Brightfield micrographs of hMSCs showed normal morphology. Scale bar, 50 µm. (B) Flow cytometry demonstrated that sorted hMSCs uniformly expressed the MSC-specific surface markers CD73, CD90 and CD105. (C) qRT-PCR analysis demonstrated the deletion of *RAP1* at the transcriptional level in *RAP1*^*−/−*^ hMSCs by primers P8 and P9. Data were presented as the mean ± SEM, *n* = 3. ***P < 0.001. (D) Immunofluorescence micrographs of RAP1 in WT and *RAP1*^*−/−*^ hMSCs. Scale bar, 10 µm. (E) Western blotting analysis demonstrated the absence of RAP1 in *RAP1*^*−/−*^ hMSCs. β-Actin was used as a loading control. (F) Characterization of the differentiation potential of hMSCs. Toluidine blue O, Oil Red O and Von Kossa staining were used to detect chondrocytes, adipocytes and osteoblasts, respectively. Scale bar, 200 µm. (G) Cell growth curves showed the enhanced proliferation ability of *RAP1*^*−/−*^ hMSCs. (H) Immunostaining of the proliferation marker Ki67 in WT and *RAP1*^*−/−*^ hMSCs. EP (early passage) and LP (late passage) represented P2 and P9, respectively. Scale bar, 50 µm. Data were presented as the mean ± SEM, *n* = 6. NS, not significant, ***P < 0.001. (I) Clonal expansion analysis of WT and *RAP1*^−/−^ hMSCs. EP and LP represented P2 and P9, respectively. Scale bar, 50 µm. Data were presented as the mean ± SEM, *n* = 3. **P < 0.01, ***P < 0.001. (J) Cell cycle analysis of WT and *RAP1*^−/−^ hMSCs (LP). Data were presented as the mean ± SEM, *n* = 3. NS, not significant, *P < 0.05, ***P < 0.001. (K) SA-β-gal staining of WT and *RAP1*^−/−^ hMSCs. EP and LP represented P2 and P9, respectively. Scale bar, 50 μm. Data were presented as the mean ± SEM, *n* = 6. NS, not significant, ***P < 0.001. (L) Western blotting analysis showed decreased expression of P16 and P21 in *RAP1*^−/−^ hMSCs (LP). β-Actin was used as a loading control. (M) Photon flux from TA muscle implanted with WT (left) and *RAP1*^−/−^ (right) hMSCs (LP) expressing luciferase. Data were presented as the mean ± SEM of *RAP1*^−/−^ to WT ratios (Log_2_(fold change)), *n* = 5. **P < 0.01, ***P < 0.001. (N) Identification of CNVs by whole-genome sequencing analysis in WT and *RAP1*^−/−^ hMSCs (EP) showed genomic integrity in *RAP1*^−/−^ hMSCs

To elucidate the role of RAP1 in human somatic stem cells, we first differentiated wild type (WT) and *RAP1*^−/−^ hESCs into hMSCs. Compared to WT hMSCs, *RAP1*^−/−^ hMSCs exhibited normal morphology and expressed hMSC-specific surface markers including CD73, CD90 and CD105 (Fig. 2A and 2B). The absence of RAP1 mRNA and protein was confirmed by qRT-PCR, immunofluorescence and Western blotting (Fig. 2C–E). In addition, *RAP1*^−/−^ hMSCs maintained the ability to differentiate into chondrocytes, adipocytes and osteoblasts (Fig. 2F).

Higher proliferation ability of *RAP1*^−/−^ hMSCs was observed through serial passaging relative to that of WT hMSCs, in which growth arrest occurred at passage 9; by comparison, *RAP1*^−/−^ hMSCs kept growing until passage 17 (Fig. 2G). Ki67 immunostaining and clonal expansion formation assays further confirmed improved proliferation ability of *RAP1*^−/−^ hMSCs (Fig. 2H and 2I). Consistently, *RAP1*^−/−^ hMSCs had more cells in S phase relative to WT hMSCs (Fig. 2J). Furthermore, *RAP1*^−/−^ hMSCs exhibited lower rate of senescence-associated β-galactosidase (SA-β-gal)-positive cells and less abundant senescence-associated proteins at late passage (Fig. 2K and 2L). In line with improved proliferation and delayed senescence, *RAP1*^−/−^ hMSCs were resistant to *in vivo* attrition after being implanted into the tibialis anterior (TA) muscle of nude mice (Fig. 2M). Genome-wide copy number variation (CNV) analysis demonstrated high genomic integrity in *RAP1*^−/−^ hMSCs (Fig. 2N), excluding the possibility that the cellular phenotypes resulted from chromosomal aberrations. Taken together, these results indicate that RAP1 deficiency promoted proliferation and slowed senescence in hMSCs.

### RAP1 deficiency led to telomere elongation in hMSCs

**Figure 3:**
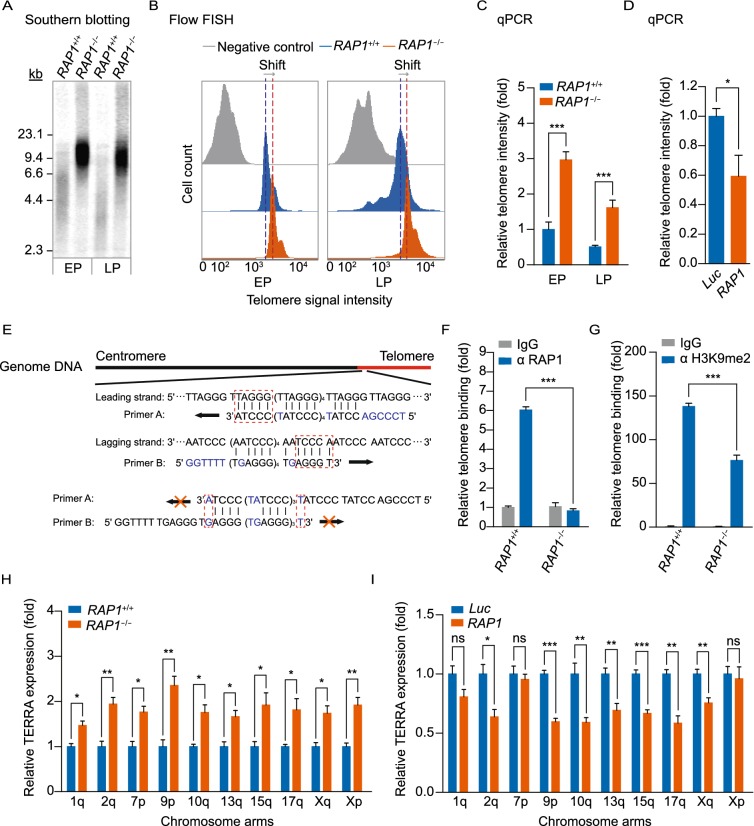
*RAP1*^−/−^ hMSCs contained longer telomeres. (A) Terminal restriction fragment analysis of WT and *RAP1*^−/−^ hMSCs by Southern blotting demonstrated longer telomeres in *RAP1*^−/−^ hMSCs. EP and LP represented P2 and P9, respectively. (B) Telomere length analysis of WT and *RAP1*^−/−^ hMSCs by flow FISH showed a right shift of the telomere signal in *RAP1*^−/−^ hMSCs. EP and LP represented P2 and P9, respectively. (C) Telomere length analysis of WT and *RAP1*^−/−^ hMSCs by qPCR. EP and LP represented P2 and P9, respectively. Data were presented as the mean ± SEM, *n* = 3. ***P < 0.001. (D) Telomere length analysis of *RAP1*^−/−^ hMSCs expressing luciferase (*Luc*) or *RAP1* by qPCR. Data were presented as the mean ± SEM, *n* = 3. *P < 0.05. (E) Schematic representation of the primers designed for telomere detection explaining how primer A and B specifically amplified telomeres rather than forming primer dimers. (F) ChIP-PCR analysis of RAP1 enrichment at the telomeres in WT and *RAP1*^−/−^ hMSCs (EP). Data were presented as mean ± SEM, *n* = 3. ***P < 0.001. (G) ChIP-PCR analysis of H3K9me2 enrichment at the telomeres in WT and *RAP1*^−/−^ hMSCs (EP) indicated a looser telomere structure in *RAP1*^−/−^ hMSCs. Data were presented as the mean ± SEM, *n* = 3. ***P < 0.001. (H) qRT-PCR analysis showed that *RAP1*^−/−^ hMSCs (EP) expressed more TERRA. Data were presented as the mean ± SEM, *n* = 3. NS, not significant, *P < 0.05, **P < 0.01. (I) TERRA analysis of *RAP1*^−/−^ hMSCs expressing luciferase or *RAP1* by qRT-PCR. Data were presented as the mean ± SEM, *n* = 3. NS, not significant, *P < 0.05, **P < 0.01, ***P < 0.001

Given the known role of RAP1 as a telomere binding protein, we next evaluated telomeric alterations in *RAP1*^−/−^ hMSCs. Compared with WT hMSCs, *RAP1*^−/−^ hMSCs exhibited longer telomeres, which was verified by multiple methods, including Southern blotting, flow FISH (flow cytometry and FISH) and genomic qPCR (Fig. 3A–C). The elongated telomeres were partially rescued by the re-introduction of RAP1 in *RAP1*^−/−^ hMSCs (Fig. 3D). A chromatin immunoprecipitation (ChIP)-PCR assay further demonstrated the association of RAP1 with the telomeres in WT hMSCs, rather than in RAP1-deficient hMSCs (Fig. 3E and 3F). Meanwhile, RAP1 deficiency resulted in less enrichment of H3K9me2, a heterochromatin marker, at the telomeres in hMSCs (Fig. 3G). Consistent with a previous observation that the heterochromatin status of telomeres affects TERRA expression (Arnoult et al., 2012), TERRA transcripts were upregulated in *RAP1*^−/−^ hMSCs (Fig. 3H), which was reversible upon addition of exogenous RAP1 (Fig. 3I). However, RAP1-deficient hMSCs exhibited no effect on the resistance to kinds of DNA damage stressors (Fig. S1A and S1B). Taken together, these results suggest that RAP1 counteracted telomere length in hMSCs.

### RAP1 deficiency downregulated *RELN* in hMSCs

**Figure 4:**
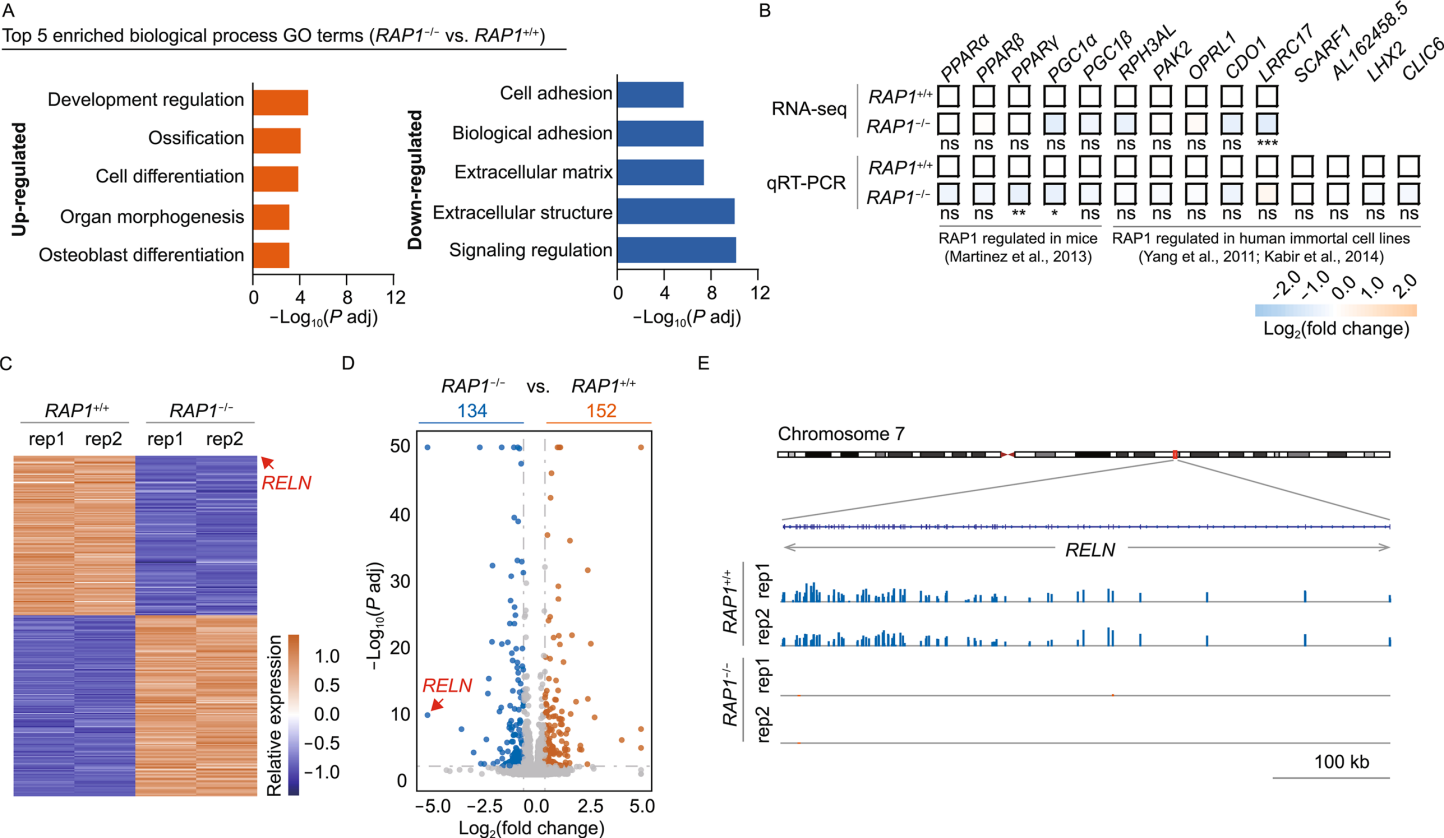

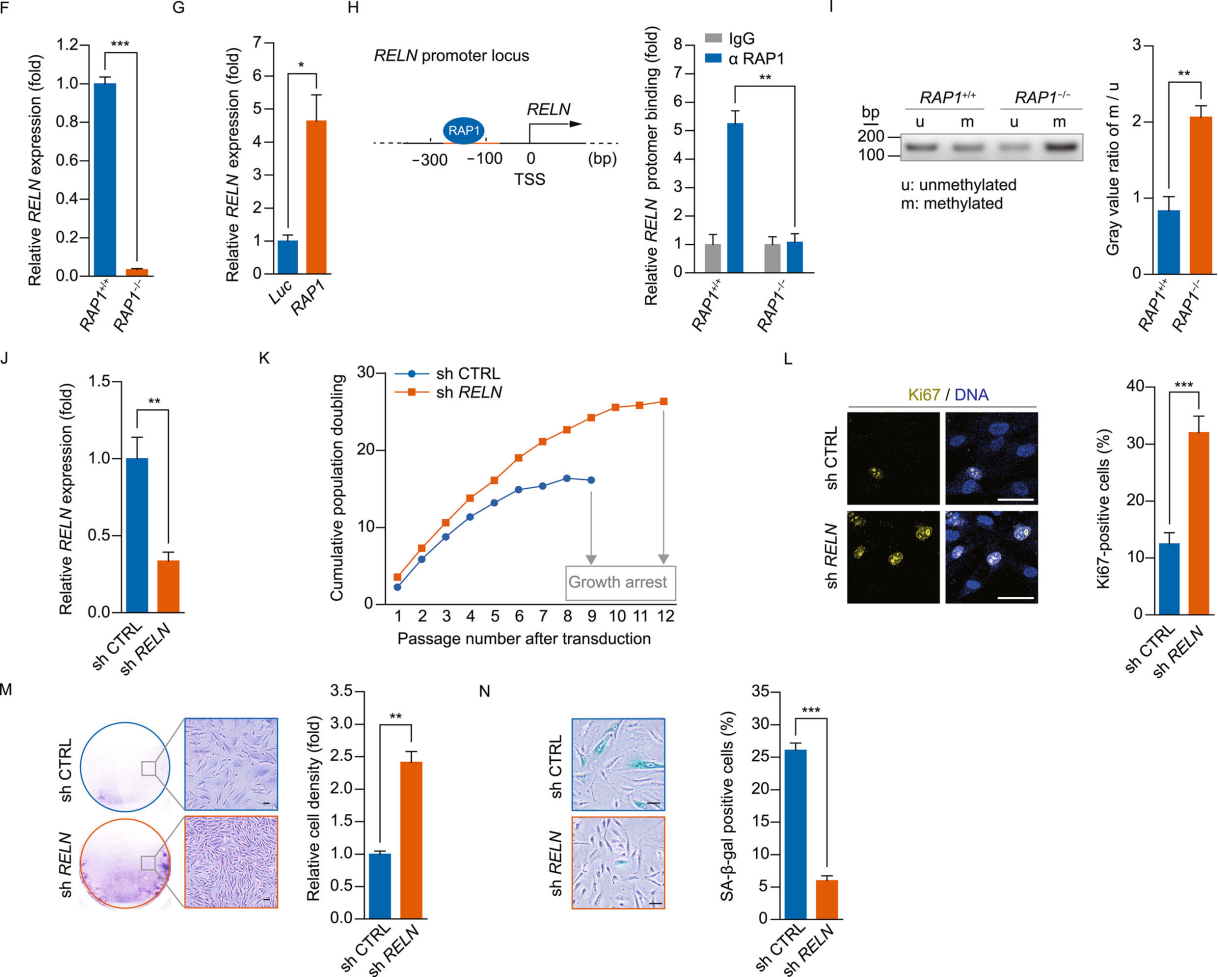
RAP1 regulated the expression of *RELN* in hMSCs. (A) Biological process GO enrichment analysis of differentially expressed genes in *RAP1*^−/−^ vs. WT hMSCs (EP). The top 5 enriched biological process GO terms were shown. (B) Heatmap of reported genes regulated by RAP1 in mice or human immortalized cell lines. Relative expression of indicated genes tested by RNA-seq and qRT-PCR demonstrated comparable levels in WT and *RAP1*^−/−^ hMSCs (EP). *n* = 3 (qRT-PCR) or 2 (RNA-seq). NS, not significant, *P_(qRT-PCR)_ or *P* adj _(RNA-seq)_ < 0.05, **P or *P* adj < 0.01, ***P or *P* adj < 0.001. (C) Heatmap of differentially expressed genes in *RAP1*^−/−^ vs. WT hMSCs (EP). *RELN* was labeled by an arrow. (D) Volcano plot of differentially expressed genes in *RAP1*^−/−^ vs. WT hMSCs (EP). *RELN* was labeled by an arrow. (E) Transcriptional signals of *RELN* in *RAP1*^−/−^ vs. WT hMSCs (EP). Transcriptional signals were normalized by RPKM at a bin size of 10 bp. (F) qRT-PCR analysis verified that *RELN* was downregulated in *RAP1*^−/−^ hMSCs (EP). Data were presented as the mean ± SEM, *n* = 3. ***P < 0.001. (G) qRT-PCR analysis demonstrated that expressing *RAP1* partially rescued the expression of *RELN* in *RAP1*^−/−^ hMSCs. Data were presented as the mean ± SEM, *n* = 3. *P < 0.05. (H) Diagram showing that RAP1 bound immediately upstream of the TSS of *RELN* in WT hMSCs (EP). ChIP-PCR analysis showed that RAP1 was enriched at the *RELN* promoter region. Data were presented as the mean ± SEM, *n* = 3. **P < 0.01. (I) Methylation-specific PCR analysis of the *RELN* promoter in WT and *RAP1*^−/−^ hMSCs (EP) demonstrated hypermethylation of the *RELN* promoter in *RAP1*^−/−^ hMSCs. Data were presented as the mean ± SEM, *n* = 3. **P < 0.01. (J) qRT-PCR analysis demonstrated that *RELN* shRNA (sh *RELN*) effectively decreased the mRNA of *RELN* than control shRNA (sh CTRL). Data were presented as the mean ± SEM, *n* = 3. **P < 0.01. (K) Cell growth curves showed that reducing the expression of *RELN* enhanced the proliferation ability of WT hMSCs. (L) Immunostaining of the proliferation marker Ki67 in WT hMSCs transfected with control or *RELN* shRNA. Scale bar, 50 μm. Data were presented as the mean ± SEM, *n* = 6. ***P < 0.001. (M) Clonal expansion analysis of WT hMSCs transfected with control or *RELN* shRNA. Scale bar, 50 μm. Data were presented as the mean ± SEM, *n* = 3. **P < 0.01. (N) SA-β-gal staining of WT hMSCs transfected with control or *RELN* shRNA. Scale bar, 50 μm. Data were presented as the mean ± SEM, *n* = 6. ***P < 0.001

To investigate whether any telomere-independent function of RAP1 was present, we carried out genome-wide RNA sequencing (RNA-seq) analysis in WT and *RAP1*^−/−^ hMSCs. In *RAP1*^−/−^ hMSCs, there were 134 downregulated genes and 152 upregulated genes comparing to those in WT hMSCs (*P* adj < 0.05 and |Log_2_(fold change)| > 0.5) (Tables S1 and S2). Biological process gene ontology (GO-BP) enrichment analysis revealed that the upregulated genes were mainly associated with development and cellular differentiation, and the downregulated genes with cell adhesion and extracellular matrix organization (Fig. 4A). In addition, known RAP1 target genes in mammals were mostly unaffected in RAP1-deficient hMSCs (Martinez et al., 2010, 2013; Yang et al., 2011; Kabir et al., 2014) (Fig. 4B). Notably, we found that *RELN*, a negative regulator of proliferation (Sato et al., 2006; Kundakovic et al., 2007; Schulze et al., 2017), was dramatically downregulated in RAP1-deficient hMSCs (Fig. 4C–F). Furthermore, the expression level of *RELN* was partially rescued by exogenously expressed RAP1 (Fig. 4G). These data suggest that RAP1 positively regulated *RELN* expression in hMSCs.

We further found that RAP1 was associated with the *RELN* promoter by ChIP analysis (Fig. 4H). Considering that the transcription of *RELN* depends on the methylation status of its promoter (Sato et al., 2006; Kundakovic et al., 2007, 2009; Lintas et al., 2016; Nabil Fikri et al., 2017), we next evaluated changes in the methylation status of the *RELN* promoter upon RAP1 deletion. In line with decreased *RELN* expression, *RELN* promoter was hypermethylated in *RAP1*^−/−^ hMSCs (Fig. 4I). These data suggest that the binding of RAP1 to *RELN* promoter in WT hMSCs was associated with a lower methylation level at this region, which may facilitate the transcription of *RELN*. To determine whether downregulation of *RELN* partially contributed to improved proliferation in RAP1-deficient hMSCs, we knocked down *RELN* via a lentiviral shRNA vector (Fig. 4J). Downregulation of *RELN* promoted proliferation ability and delayed senescence in WT hMSCs (Fig. 4K–N). Taken together, these data indicate that RAP1 regulated the proliferation and senescence of hMSCs at least in part via the epigenetic derepression of *RELN* expression.

Since *RELN* encodes Reelin, which is a typical marker of preplate/Cajal-retzius cells in the brain and plays an important role in the development of the nervous system (Lancaster et al., 2013; Lancaster and Knoblich, 2014; Sekine et al., 2014; Ishii et al., 2016), we performed cerebral differentiation using a 3D culture based procedure (Fig. S2A) and achieved organoid-like cerebral structures by using WT and *RAP1*^−/−^ hESCs. As expected, the resultant organoid-like cerebral structures by *RAP1*^−/−^ hESCs lacked RAP1 expression and the layer containing preplate/Cajal-retzius cells marked by Reelin (Fig. S2B–E), further supporting a role of RAP1 in regulating *RELN* expression.

### RAP1 had no effect on the proliferation and senescence of hNSCs

**Figure 5:**
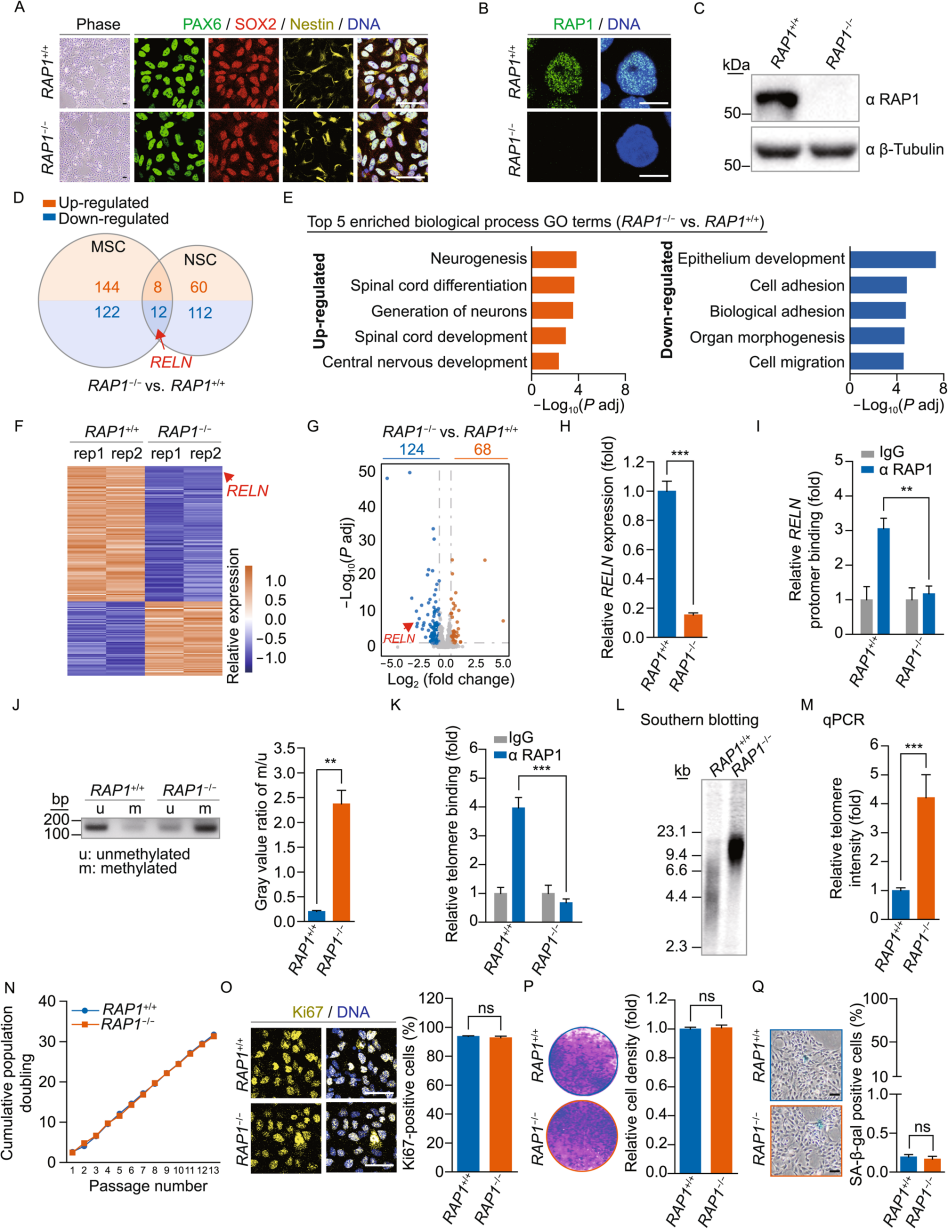
RAP1 deficiency did not influence the proliferation or senescence of hNSCs. (A) Brightfield and immunofluorescence micrographs of WT and *RAP1*^−/−^ hNSCs showed normal morphology and expression of the NSC markers PAX6, SOX2 and Nestin. Scale bar, 50 μm. (B) Immunofluorescence micrographs of RAP1 in WT and *RAP1*^−/−^ hNSCs. Scale bar, 10 μm. (C) Western blotting analysis demonstrated the absence of RAP1 in *RAP1*^−/−^ hNSCs. β-Tubulin was used as a loading control. (D) Venn diagram showing differentially expressed genes in *RAP1*^−/−^ vs. WT hMSCs and hNSCs. *RELN* was labeled by an arrow. (E) Biological process GO enrichment analysis of differentially expressed genes in *RAP1*^−/−^ vs. WT hNSCs. The top 5 enriched biological process GO terms were shown. (F) Heatmap of differentially expressed genes in *RAP1*^−/−^ vs. WT hNSCs. *RELN* was labeled by an arrow. (G) Volcano plot of differentially expressed genes in *RAP1*^−/−^ vs. WT hNSCs. *RELN* was labeled by an arrow. (H) qRT-PCR analysis verified that *RELN* was downregulated in *RAP1*^−/−^ hNSCs. Data were presented as the mean ± SEM, *n* = 3. ***P < 0.001. (I) ChIP-PCR of RAP1 enrichment at the *RELN* promoter region in WT and *RAP1*^−/−^ hNSCs. Data were presented as the mean ± SEM, *n* = 3. **P < 0.01. (J) Methylation-specific PCR analysis of the *RELN* promoter in WT and *RAP1*^−/−^ hNSCs demonstrated hypermethylation of the *RELN* promoter in *RAP1*^−/−^ hNSCs. Data were presented as the mean ± SEM, *n* = 3. **P < 0.01. (K) ChIP-PCR analysis of RAP1 enrichment at the telomeres in WT and *RAP1*^−/−^ hNSCs. Data were presented as the mean ± SEM, *n* = 3. ***P < 0.001. (L) Terminal restriction fragment analysis of WT and *RAP1*^−/−^ hNSCs by Southern blotting demonstrated the elongated telomere length in *RAP1*^−/−^ hNSCs. (M) Telomere length analysis of WT and *RAP1*^−/−^ hNSCs by qPCR. Data were presented as the mean ± SEM, *n* = 3. ***P < 0.001. (N) Cell growth curves of WT and *RAP1*^−/−^ hNSCs showed comparable proliferation ability of WT and *RAP1*^−/−^ hNSCs. (O) Immunostaining of the proliferation marker Ki67 in WT and *RAP1*^−/−^ hNSCs. Scale bar, 50 μm. Data were presented as the mean ± SEM, *n* = 6. NS, not significant. (P) Clonal expansion analysis of WT and *RAP1*^−/−^ hNSCs. Data were presented as the mean ± SEM, *n* = 3. NS, not significant. (Q) SA-β-gal staining of WT and *RAP1*^−/−^ hNSCs. Scale bar, 50 μm. Data were presented as the mean ± SEM, *n* = 6. NS, not significant

To test whether RAP1 regulated cell proliferation and senescence in a cell type-specific manner, we differentiated WT and *RAP1*^−/−^ hESCs into hNSCs. *RAP1*^−/−^ hNSCs demonstrated normal neural progenitor morphology, expressed the NSC-specific markers PAX6, SOX2 and Nestin (Fig. 5A), and maintained neuronal differentiation ability (Fig. S3A). Ablation of RAP1 protein in *RAP1*^−/−^ hNSCs was verified by immunofluorescence and Western blotting (Fig. 5B and 5C). RNA-seq analysis revealed a total of 124 downregulated genes and 68 upregulated genes in *RAP1*^−/−^ hNSCs comparing to those in WT hNSCs (*P* adj < 0.05 and |Log_2_(fold change)| > 0.5) (Tables S3 and S4). However, among the 192 differentially expressed genes between *RAP1*^−/−^ and WT hNSCs and 286 differentially expressed genes between *RAP1*^−/−^ and WT hMSCs, only 20 genes were overlapped (Fig. 5D). Notably, *RELN* was still one of the most downregulated genes in *RAP1*^−/−^ hNSCs (Fig. 5E–H). Consistently, silencing of *RELN* expression in *RAP1*^−/−^ hNSCs was associated with the hypermethylation at the *RELN* promoter (Fig. 5I and 5J). Similar to RAP1-deficient hMSCs, depletion of RAP1 in hNSCs resulted in longer telomeres (Fig. 5K–M). However, no proliferation-promoting effect was observed in *RAP1*^−/−^ hNSCs (Fig. 5N–Q). Additionally, the *in vitro* migration ability of hNSCs was also comparable between WT and *RAP1*^−/−^ hNSCs (Fig. S3B). Therefore, RAP1 deficiency itself was insufficient to promote proliferation in hNSCs despite its marked effects on *RELN* expression and telomere length.

## DISCUSSION

**Figure 6:**
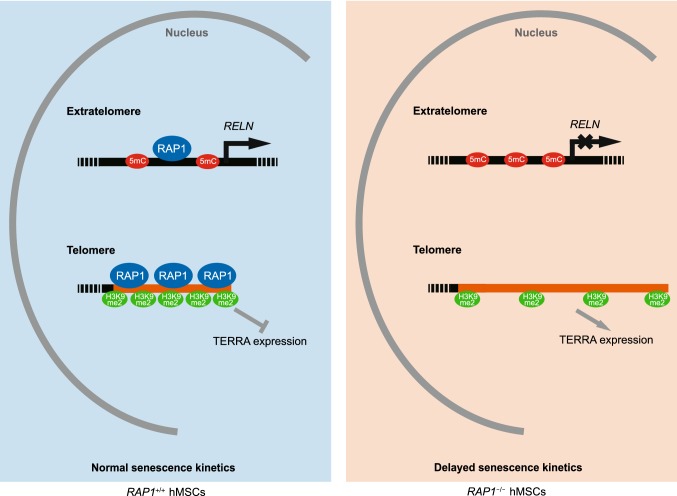
A proposed model showing how RAP1 regulates hMSC homeostasis

With the aid of the CRISPR/Cas9-mediated gene-editing technique, our study revealed for the first time that RAP1 negatively regulated telomere length as a telomere-binding protein and positively regulated the expression of *RELN* as a potential epigenetic regulator in both hMSCs and hNSCs. Interestingly, RAP1 functioned as a proliferation/senescence regulator only in hMSCs (Fig. 6), but not in hNSCs. Thus, our results provide an important evidence that RAP1 may play a role in regulating human stem cell homeostasis in a lineage-specific manner.

The effects of RAP1 in regulating cell proliferation have been controversial (Table S5). RAP1-knockout mice exhibit normal growth and lifespan (Martinez et al., 2010, 2013, 2016; Sfeir et al., 2010). However, RAP1 deficiency decreased the proliferation of the human hepatocellular carcinoma cell line HepG2 and non-small cell lung cancer cell line A549 (Zha et al., 2014; Xiao et al., 2017), but not that of the human fibrosarcoma cell line HT1080 (Kabir et al., 2014) or mouse MSCs (Ding et al., 2018). It is therefore likely that the effect of RAP1 in growth regulation is species or cell type-specific. In this study, we generated RAP1-knockout hESCs and obtained their hMSC and hNSC derivatives via directed differentiation. With these cell models, we observed a lineage-specific effect of RAP1 in regulating stem cell senescence despite the altered telomere length and *RELN* expression. It is possible that specific factors in RAP1-knockout hNSCs may compensate for the effect of RAP1 deficiency on proliferation regulation. The cell type-specific effects could also be explained by the notion that RAP1 regulates distinct sets of genes in hMSCs and hNSCs. For instance, among the most upregulated genes in *RAP1*^−/−^ hMSCs, several are implicated in cell proliferation, such as *S100A6* (Donato et al., 2017), *PLXNA4* (Di Palma et al., 2013), *MAGED4B* (Ferrara-Romeo et al., 2018) and *PAX8* (Di Palma et al., 2013). However, none of those gene expression changes were observed in *RAP1*^−/−^ hNSCs.

Although RAP1 is a well-known telomere-binding protein, the mechanism by which RAP1 regulates telomere length remains controversial. RAP1 has been reported to protect against telomere attrition in tail skin and small intestine in mice (Martinez et al., 2010, 2016), but negatively regulates telomere length in human immortalized cells (Li and de Lange, 2003; O’Connor et al., 2004; Kim et al., 2017). By contrast, Kabir et al. knocked out the exon 2 of *RAP1* by TALEN technique in multiple human immortalized cell lines and observed no effect of RAP1 deficiency on telomere length or any other telomere-related parameters (Kabir et al., 2014). Given that the telomere biology of rodent cells and human immortalized cell lines is substantially different from that of human diploid cells (Calado and Dumitriu, 2013; Reddel, 2014), it is necessary to clarify the role of RAP1 in human diploid cells in a physiological context. Therefore, the RAP1-deficient human diploid cells that we established here represent superior models for gaining a better understanding of RAP1-mediated telomere regulation in human adult stem cells. We observed that RAP1 deficiency elongated telomeres in both hMSCs and hNSCs, which is consistent with observations in lower eukaryotes (Kanoh and Ishikawa, 2001) and the “shelterin counting mechanism” theoretical model (Smogorzewska and de Lange, 2004). Our data also demonstrated that RAP1 negatively regulated TERRA expression, which is in accordance with a study in lower eukaryotes rather than human immortalized cell lines (Kabir et al., 2014; Nanavaty et al., 2017). Thus, our data not only highlight the importance of using human stem cell models to study the role of RAP1 in telomere regulation, but also support a conserved function for RAP1 from yeast to human.

In addition to its telomere-dependent function, mammalian RAP1 plays a role in regulating gene expression. However, most past studies have failed to unravel the underlying mechanisms (Yang et al., 2011; Kabir et al., 2014; Martinez et al., 2016) with the exception of one study, in which RAP1 protected mice from obesity by promoting the transcription of *Pparα* and *Pgc1α* by directly binding to the upstream regulatory regions of these genes (Martinez et al., 2010, 2013). In our study, RAP1 bound to the promoter of *RELN* and kept it from being hypermethylated in hMSCs and hNSCs, which facilitated the transcription of *RELN* and accounted at least in part for the delayed senescence in hMSCs. These results provide a novel explanation for how RAP1 regulates the expression of its target genes at the epigenetic level. While how RAP1 regulates DNA methylation is unclear, which warrants further investigation, it is possible that RAP1 *per se* functions as a demethylase, promotes the activity of certain DNA demethylases, or prevents certain DNA methylases from acting on *RELN* promoter; it is also possible that RAP1 affects the function of DNA methylases or demethylases by changing chromatin conformation nearby.

Emerging evidences support the notion that stem cell attrition is closely associated with aging and various age-related diseases (Lopez-Otin et al., 2013; Liu et al., 2014; Goodell and Rando, 2015; Zhang et al., 2015; Kubben et al., 2016; Pan et al., 2016; Yang et al., 2017; Wang et al., 2018b; Wu et al., 2018). Here, we highlight an important role for RAP1 in regulating hMSC aging, potentially through both telomere-dependent and telomere-independent functions. These new findings may open new avenues of research to better understand the mechanisms of human stem cell attrition and identify new strategies to treat aging-associated disorders.

## MATERIALS AND METHODS

### Cell culture

Human H9 (WT) and *RAP1*^−/−^ hESCs were cultured on mouse embryonic fibroblasts (MEFs) inactivated by mitomycin C (Selleck) in hESC medium (DMEM/F12 (Invitrogen) supplemented with 20% Knockout Serum Replacement (Invitrogen), 2 mmol/L GlutaMAX (Invitrogen), 0.1 mmol/L non-essential amino acids (NEAA, Invitrogen), 1% penicillin/streptomycin (Invitrogen), 55 μmol/L β-mercaptoethanol (Invitrogen) and 10 ng/mL FGF-2 (Joint Protein Central)) (Fu et al., 2016) or on Matrigel (BD Biosciences) in mTeSR medium (STEMCELL Technology). hMSCs were cultured on 0.1% gelatin (Sigma) in hMSC culture medium (MEMα (Invitrogen) supplemented with 10% fetal bovine serum (FBS, Gemcell, Cat. No. 100-500, lot. No. A77E01F), 2 mmol/L GlutaMAX, 0.1 mmol/L NEAA, 1% penicillin/streptomycin and 1 ng/mL FGF-2). hNSCs were cultured in plates coated by Matrigel in neural stem cell maintenance medium (50% Advanced DMEM/F12 (Invitrogen) and 50% Neurobasal (Invitrogen), supplemented with 1% N2 (Invitrogen), 2% B27 (Invitrogen), 2 mmol/L GlutaMAX, 0.1 mmol/L NEAA, 1% penicillin/streptomycin, 10 ng/mL human leukemia inhibitory factor (Millipore), 3 μmol/L CHIR99021 (Selleck) and 2 μmol/L SB431542 (Selleck)). Additionally, 10 μmol/L ROCK inhibitor Y-27632 (Sigma) was added prior to re-plating for each passage before passage 6. HEK293T cells were cultured in 293T culture medium (high glucose DMEM (HyClone) supplemented with 10% FBS (Gemini), 2 mmol/L GlutaMAX, 0.1 mmol/L NEAA and 1% penicillin/streptomycin). All cells were cultured in an incubator at 37 °C with 5% CO_2_.

### Generation of RAP1-knockout (*RAP1*^−/−^) hESCs via the CRISPR/Cas9 technique

Gene editing via the CRISPR/Cas9 technique was slightly modified from a previously published protocol (Wang et al., 2018b). The *RAP1* guide RNA (gRNA) 5′-TGGGTGAATGAGCACGTCCT-3′ was cloned into the gRNA-Cloning Vector (Addgene, #41824). The donor vector contained homology arms and a neo cassette flanked with two FRT sites for HR (Pan et al., 2016). 5 × 10^6^ H9 ESCs pretreated with 10 μmol/L Y-27632 were mixed with three plasmids (sgRNA, donor and Cas9 expression vectors)  and then electroporated. Post-electroporated cells were plated on G418-resistant MEF feeders with 10 μmol/L Y-27632. Once hESC clones formed, cell screening was performed by the addition of 100 μg/mL G418 (Sigma). The neo cassette was removed as previously described (Duan et al., 2015) . The clones were picked for expansion and verified by genomic PCR. The primers for the HR donor vector construction were 5′-CTATAGGGCGAATTGGGCCC AGCCTCTATTACCGTCTCTTGTCTGTTGCAT-3′ (forward) and 5′-CTGGCGGCCGCTCGAGGGCCACGTACCACAATCCACCAATATACCAT-3′ (reverse) for the left arm and 5′-TTACTAGTGGATCCGAGCTCTGGAAAATGGGACTGATCTGGGCTTCAGAC-3′ (forward) and 5′-ATTACGCCAAGCTTGGTACCTCACCACATCTCCAATACCCACCAATGCCTA-3′ (reverse) for the right arm. The primers for clone identification were P1: 5′-TTGGCAAAAGTCAATACAATGGGTAATATCCAAAG-3′ (forward), P2: 5′-GGGCATTTTGACAATATCTGATGACATTTATAACG-3′ (forward). P3: 5′-AAAACTCCCTCTTGCTGCCCCTTTGT-3′ (forward), P4: 5′-GTGGATTGTGGTACGTGGCCCAGATCTGCC-3′ (forward), P5: 5′-TAACATACCACAACCTCCTCAAACTCCCGG-3′ (reverse), P6: 5′-TGTCCTGCCAAAAACTAAAAGCTTTGTGA-3′ (reverse), and P7: 5′-TTTGACTTCACTCTCAAGACTGTAAGCTCCT -3′ (reverse).

### Generation and characterization of hMSCs

hMSCs were derived from hESCs as described previously (Pan et al., 2016). In brief, embryoid bodies (EBs) first formed from hESC clones in an ultralow attachment 6-well plate (Corning) in low FGF-2 hESC medium and then were transferred to a plate coated by Matrigel in hMSC differentiation medium (hMSC culture medium supplemented with additional 9 ng/mL FGF-2 and 5 ng/mL TGF-β (HumanZyme)). After 7 to 10 days, the cells became confluent and were reseeded into dishes coated by gelatin in hMSC culture medium. CD73, CD90 and CD105 tri-positive cells were sorted as hMSCs with the aid of flow cytometry. The following antibodies were used: anti-CD73-PE (BD Biosciences, 550257), anti-CD90-FITC (BD Biosciences, 555595) and anti-CD105-APC (eBioscience, 17-1057-42). The differentiation abilities of hMSCs were tested by futher differentiation into chondrocytes, adipocytes and osteoblasts (Liu et al., 2014) detected by toluidine blue (chondrocytes), oil red O (adipocytes) and von Kossa (osteoblasts) staining, respectively.

### Generation and characterization of hNSCs

hNSCs were derived from hESCs as described previously (Duan et al., 2015). Briefly, hESCs were cultured on MEF feeders in neural induction medium-1 (neural stem cell maintenance medium supplemented with 1 μmol/L CHIR99021, 1 μmol/L SB431542, 2 μmol/L dorsomorphin (Sigma) and 0.1 μmol/L Compound E (EMD Chemicals Inc.)) for two days and then neural induction medium-2 (neural induction medium-1 without dorsomorphin) for five days. The cells were subsequently cultured in plates coated by Matrigel in neural stem cell maintenance medium. The hNSC markers PAX6, SOX2 and Nestin were detected by immunofluorescence microscopy. The differentiation ability of hNSCs towards neurons was evaluated by MAP2 and TUJ1 immunostaining (Zhang et al., 2018).

### Generation of organoid-like cerebral structures

Organoid-like cerebral structures were differentiated from hESCs as described previously (Lancaster et al., 2013; Lancaster and Knoblich, 2014). Briefly, hESCs cultured on MEF feeders were digested into single cells. EBs were formed from 2 × 10^4^ cells in a U-bottom ultralow attachment 96-well plate (Corning) in low FGF-2 hESC medium supplemented with 50 μmol/L Y-27632. After four days, FGF-2 and Y-27632 were deprived for another two days. Then, the EBs were transferred to ultralow attachment 24-well plates (Corning) in neural induction medium (DMEM/F12 supplemented with 1% N2, 2 mmol/L GlutaMAX, 0.1 mmol/L NEAA, 1% penicillin/streptomycin and 1 μg/mL heparin (Selleck)) for four days. The EBs were packaged into Matrigel droplets and further cultured in 6-well plates in cerebral organoid differentiation medium (50% DMEM/F12 and 50% Neurobasal, with additional 0.5% N2, 1% B27 without vitamin A (Invitrogen), 2 mmol/L GlutaMAX, 0.05 mmol/L NEAA, 1% penicillin/streptomycin, 2.75 μg/mL insulin (Sigma) and 50 μmol/L β-mercaptoethanol (Sigma)) for four days. Then, the plates were placed on an orbital shaker (shaking at 85 rpm) installed in the incubator, and B27 without vitamin A was replaced by normal B27. After approximately 40 days, the structures were fixed by 4% (*w*/*v*) paraformaldehyde for cryosectioning and immunostaining or collected directly for RNA extraction.

### Lentivirus production

Lentiviruses were expressed and purified as described previously (Duan et al., 2015). To construct the RAP1-overexpression lentiviral vector, the cDNA of flag-RAP1 was amplified by PCR with the primers 5′-CCGCTCGAG ATGGACTACAAGGACGACGACGACAAGGGCGCGGAGGCGATGGATTTGGG-3′ (forward) and 5′-CGACGCGTTTATTTCTTTCGAAATTCAATCCTCCGAGC-3′ (reverse), cleaved by *Xho*I (NEB) and *Mlu*I (NEB), and cloned into the pLE4 vector (a kind gift from Doctor Tomoaki Hishida) pre-cleaved by *Xho*I and *Mlu*I. To generate the lentiviral vector encoding shRNA targeting *RELN*, the annealed fragment from the oligos 5′-CGCGT GCACGGATGAAAGGAGTCTTATTCAAGAGATAAGACTCCTTTCATCCGTGCTTTTTTGGAAAT-3′ (forward) and 5′-CGATTTCCAAAAAA GCACGGATGAAAGGAGTCTTATCTCTTGAATAAGACTCCTTTCATCCGTGCA-3′ (reverse) was phosphorylated by T4 Polynucleotide Kinase (NEB) and cloned into the pLVTHM vector pre-cleaved by* Cla*I (NEB) and* Mlu*I. For lentiviral packaging, HEK293T cells were cultured up to 95% confluency and then co-transfected with the overexpression or shRNA vectors, along with psPAX2 (Addgene, #12260) and pMD2.G (Addgene, #12259). Two days later, the culture medium was collected and concentrated by ultracentrifugation at 4 °C. The lentiviral particles were used for transduction with 4 µg/mL polybrene.

### Clonal expansion assay

Two thousand cells were seeded in a well of a 12-well plate (Corning) and cultured for approximately 10 days. Only for hESCs, 10 μmol/L Y-27632 was added on the first day of cell passaging and removed after 24 h. The relative colony number and relative cell integral density was calculated by ImageJ software after crystal violet staining.

### SA-β-gal staining

SA-β-gal staining was performed as described previously (Debacq-Chainiaux et al., 2009; Wu et al., 2018). In brief, cells were fixed with fixation buffer containing 2% (*w*/*v*) formaldehyde and 0.2% (*w*/*v*) glutaraldehyde for 5 min. Then, the cells were treated with staining buffer containing 1 mg/mL X-gal overnight at 37 °C. Stained cells were observed by optical microscope and the percentage of positive cells was analyzed by ImageJ software.

### Cell cycle analysis

Cell cycle analysis was performed as described previously (Wang et al., 2018b). In brief, cells were fixed by 70% precooled ethanol at least overnight and then treated with staining buffer containing 0.1% Triton X-100, 0.2 mg/mL RNase A and 0.02 mg/mL propidium iodide at 37 °C for 30 min. Then, the cells were analyzed by an LSRFortessa cell analyzer (BD), and data were measured by ModFit software.

### Cell viability analysis

MTS analysis of hMSCs was performed as described previously (Pan et al., 2016). In brief, cells at 90% confluence in 96-well plates (Corning) were treated with different stressors for 24 h. Cell vitality was measured by MTS colorimetry.

### *In vitro* cell migration analysis

Transwell assay of hNSCs was performed as described previously (Duan et al., 2015). In brief, 2.5 × 10^4^ cells were resuspended by 100 μL basal medium (50% Advanced DMEM/F12 and 50% Neurobasal) and seeded on the top of transwell (Corning) with 500 μL neural stem cell maintenance medium added to the bottom. 24 h later, the cells at the lower surface of transwells were calculated by ImageJ software after crystal violet staining.

### Genomic and bisulfite PCR

Genomic DNA was extracted by a DNA extraction kit (TIANGEN). General PCR was performed with the PrimeSTAR HS DNA Polymerase with GC Buffer Kit (TAKARA) and a 96-well thermal cycler (Applied Biosystems). Bisulfite conversion of genomic DNA was carried out with the EZ DNA Methylation-Lightning Kit (ZYMO Research). Bisulfite PCR was carried out with LA Taq Hot Start Version (TAKARA). Detection of the methylation level of the *OCT4* promoter was performed as described previously (Yu et al., 2007; Duan et al., 2015). In brief, the PCR products were recycled by the QIAquick Gel Extraction Kit (Qiagen), cloned into the T-vector (Takara) and sequenced with the universal primer M13. The methylation level of the *RELN* promoter was detected by methylation-specific PCR (Omura et al., 2008; Vincent et al., 2011) with proper primers (Sato et al., 2006). Image J software was used to calculate the gray value ratio of methylated/unmethylated bands.

### RT-PCR

For general RT-PCR, total RNA was extracted by TRIzol (Invitrogen). For RT-PCR of TERRA, total RNA was extracted by the RaPure Total RNA Micro Kit (Magen) with DNA removal on the column. Then, cDNA was synthesized with the GoScript Reverse Transcription System (Promega). The semi-quantitative RT-PCR method was the same as that for general PCR, while qRT-PCR was performed using THUNDERBIRD qPCR Mix (TOYOBO) and the CFX384 Real-Time System (BioRad). The primers for TERRA detection were described previously (Feretzaki and Lingner, 2017). Primers to detect the exon 2 of *RAP1* were P8: 5′-GGGCCAGGAGCATAAGTACC-3′ (forward) and P9: 5′-GGAGTTCTCTTATTCTGTGGTTCC-3′ (reverse) (Hohensinner et al., 2016); primers for the internal reference for general qRT-PCR, 18S rRNA, were 5′-GTAACCCGTTGAACCCCATT-3′ (forward) and 5′-CCATCCAATCGGTAGTAGCG-3′ (reverse) (Ren et al., 2017a). Other primer sequences were obtained from published articles (Takahashi et al., 2007; Yang et al., 2011; Zhang et al., 2015) or selected from PrimerBank (https://pga.mgh.harvard.edu/primerbank/).

### Western blotting

Cells were lysed in 2× SDS-sample buffer without glycerol and β-mercaptoethanol and heated at 95 °C for 10 min (Li et al., 2001). Then, the samples were quantified via a BCA (bicinchoninic acid) protein quantification assay. Generally, lysate containing 30 μg of total protein was loaded onto an SDS-PAGE gel for electrophoresis and then electrotransferred to a PVDF membrane (Millipore). After blocking with 5% (*w*/*v*) nonfat powdered milk (BBI Life Sciences), the membrane was successively incubated with primary and HRP-conjugated secondary antibodies and blotted by SuperSignal West Femto Maximum Sensitivity Substrate (Thermo Fisher). Finally, imaging and quantification were performed with the ChemiDoc XRS system (Bio-Rad) and Image Lab software. The primary antibodies used for Western blotting in this study were anti-RAP1 (Santa Cruz, sc53434), anti-P16 (BD, 550834), anti-P21 (CST, 2947s), anti-β-actin (Santa Cruz, sc69879) and anti-β-tubulin (Santa Cruz, sc5274). To confirm that RAP1 was completely knocked out in hESCs, we also used another RAP1 antibody (#765) that detects potential truncations of RAP1 (Kabir et al., 2014), which was a gift from T. de Lange.

### Telomere length analysis

Measurement of telomere length by qPCR and Southern blotting was conducted as described previously (Cawthon, 2002; Lai et al., 2016). However, capillary transfer was used. Measurement of telomere length by flow FISH was performed by imitating a 3D-FISH/immunolabeling protocol (Ren et al., 2017a). Briefly, adherent hMSCs were digested and neutralized. The cells were then incubated with 4% (*w*/*v*) paraformaldehyde, 0.4% (*v*/*v*) Triton X-100 in PBS, 100 µg/mL RNase A in PBS, and 20% (*v*/*v*) glycerol in PBS and then heated to denature genomic DNA and hybridized with Cy3-labeled telomere PNA probe (Panagene) overnight at 37 °C in a hybridization oven (UVP). The samples were measured with an LSRFortessa cell analyzer (BD), and data were analyzed by FlowJo software.

### Immunofluorescence microscopy

Cells were successively treated for 30 min with 4% (*w*/*v*) paraformaldehyde, 0.4% (*v*/*v*) Triton X-100 in PBS and dilute donkey serum (Jackson ImmunoResearch Labs), and then incubated with primary antibodies overnight at 4 °C and corresponding fluorescent secondary antibodies as well as Hoechst 33342 (Invitrogen) at room temperature for 1 h. The primary antibodies used were anti-RAP1 (Santa Cruz, sc53434), anti-OCT3/4 (Santa Cruz, sc5279), anti-SOX2 (Santa Cruz, sc17320), anti-NANOG (Abcam, ab21624), anti-TUJ1 (Sigma, T2220), anti-SMA (Sigma, A5228), anti-FOXA2 (CST, 8186), anti-Ki67 (Vector, VP-RM04), anti-PAX6 (Covance, PRB-278P), anti-Nestin (BD, 560422), anti-MAP2 (Sigma, M4403) and anti-Reelin (MBL, D223-3).

### ChIP

The ChIP assay was slightly modified from versions described previously (Dahl and Collas, 2008; Zhang et al., 2018). In brief, cells were harvested and crosslinked by 1% (*v*/*v*) formaldehyde for 15 min (RAP1) or 8 min (H3K9me2), and then termination of crosslinking was performed by 125 mmol/L glycine for 5 min. After washing with PBS, the cells were lysed and sonicated to generate DNA fragments. The product was incubated with antibody binding beads overnight at 4 °C. Then, the supernatant was removed, and the beads were decrosslinked for 2 h at 68 °C. The DNA was recycled and quantified by a qPCR assay. Antibodies for ChIP included anti-RAP1 (Santa Cruz, sc53434), anti-H3K9me2 (Abcam, ab1220) and mouse IgG (Santa Cruz, sc2025) as a negative control. The primers for the *RELN* promoter of RAP1 binding locus were 5′-CGAGCCAGCCCGAGA-3′ (forward) and 5′-GTCGTCTGCCGCCTCC-3′ (reverse).

### Animal experiments

Teratoma assay was carried out as described previously (Lensch et al., 2007). Briefly, approximately 5 × 10^6^ hESCs on feeder layers were injected into the groin cavities of NOD/SCID mice (male, 6–8 weeks). After approximately 2 months, the teratomas were taken out and analyzed by immunofluorescence staining. hMSC transplantation assay was carried out as described previously (Yang et al., 2017). In brief, cells were previously transduced with lentiviruses that express luciferase in human cells. A total of 1 × 10^6^ cells were injected into the midportion of the TA muscle of nude mice (male, 6–8 weeks). Every two days after transplantation, mice were treated with D-luciferin and then imaged by an IVIS spectrum imaging system (XENOGEN, Caliper) in AUTO mode. Animal experiments were performed with the approval of the Institute of Biophysics, Chinese Academy of Science (IBP, CAS).

### CNV identification

Genomic DNA of early-passage (P2) hMSCs was extracted by the DNeasy Blood and Tissue Kit (Qiagen).Then, the DNA was fragmented by a Covaris S220 ultrasonicator, and libraries were constructed by using the NEBNext Ultra^TM^ DNA Library Prep Kit for Illumina (NEB). The samples were clustered by the Truseq PE Cluster Kit V4 and sequenced on an Illumina Hiseq X-ten platform. For CNV analysis, paired end reads were trimmed and aligned to the UCSC hg19 human reference genome by bowtie2 software (v2.2.9) (Langmead and Salzberg, 2012). CNVs were calculated by readCounter and normalized by HMMcopy (v1.20.0) in a 500-kb window (Ha et al., 2012).

### RNA-seq library preparation and sequencing

Total RNA of early-passage hMSCs (P2) or hNSCs (P3) was extracted by TRIzol. Library construction, sequencing and processing of RNA-seq data were carried out as previously described (Geng et al., 2018; Wang et al., 2018a). Briefly, RNA integrity was first qualified by using the RNA Nano 6000 Assay Kit for the Bioanalyzer 2100 system (Agilent Technologies), and then libraries were constructed by using the NEBNext Ultra^TM^ RNA Library Prep Kit for Illumina (NEB). The samples were clustered on a cBot Cluster Generation System by TruSeq PE Cluster Kit v3-cBot-HS (Illumina) and sequenced on an Illumina Hiseq platform. Raw data were trimmed and then mapped to the UCSC hg19 human genome using hisat2 (v2.0.4) (Kim et al., 2015). The transcriptional level of each gene was counted by HTSeq (v0.6.1) (Anders et al., 2015). Differentially expressed genes were calculated by the DESeq2 R package with the cutoff Benjamini-Hochberg adjusted *P* value (*P* adj) of less than 0.05 and |Log _2_ (fold change)| of more than 0.5 (Love et al., 2014). GO-BP enrichment analysis was conducted by ToppGene (Chen et al., 2009).

### Statistical analysis

Results were presented as the mean ± SEM. Graph-Pad Prism software was used to perform a two-tailed Student’s *t*-test. Statistical significance was presented as *P values < 0.05, **P values < 0.01 and ***P values < 0.001.

## Electronic supplementary material

Below is the link to the electronic supplementary material.

Supplementary material 1 (PDF 50359 kb)

Supplementary material 2 (XLSX 23 kb)

## Data Availability

The raw data and processed data reported in this paper were deposited into the GEO database with the accession number GSE122654.
