# Supplementary material for: Telomere-dependent and telomere-independent roles of RAP1 in regulating human stem cell homeostasis
Source: Protein Cell. 2019 Feb 22;10(9):649–67. doi: 10.1007/s13238-019-0610-7 (PMC6711945; doi:10.1007/s13238-019-0610-7)
Supplement: Supplementary file 1 — Supplementary material 1 (PDF 50359 kb) [file 13238_2019_610_MOESM1_ESM.pdf]

Figure S1 RAP1 deficiency hardly influenced the resistance of hMSCs to DNA damage stressors.

(A) Cell viability of WT and *RAP1*$^{-/-}$ hMSCs (EP) treated with different DNA damage stressors was measured by MTS assay. Data were normalized to vehicle (DMSO)-treated group and were presented as the mean ± SEM, n = 3. NS, not significant.

(B) A table summarizing the dosage and effects of the DNA damage stressors.

Figure S2 RAP1 deficiency influenced the differentiation of organoid-like cerebral structures from hESCs.

(A) Diagram of organoid-like cerebral structures differentiation from hESCs. Modified from Lancaster et al. (2013).

(B) Immunofluorescence micrographs of WT and *RAP1*$^{-/-}$ organoid-like cerebral structures demonstrated reduced Reelin expression along with RAP1 deficiency. Scale bar, 50 µm.

(C) Statistical scatter diagram of relative Reelin-positive cell counts. Data were presented as mean ± SEM, n = 200. ***P < 0.001.

(D) qRT-PCR analysis demonstrated the deletion of *RAP1* at the transcriptional level in *RAP1*$^{-/-}$ cerebral-organoid like tissues by primers P8 and P9. Data were presented as the mean ± SEM, n = 3. ***P < 0.001.

(E) qRT-PCR analysis demonstrated the downregulation of *RELN* in *RAP1*$^{-/-}$ organoid-like cerebral structures. Data were presented as the mean ± SEM, n = 3. ***P < 0.001.

Figure S3 RAP1 deficiency had no effect on the neuronal differentiation and migration abilities of hNSCs.

(A) Representative brightfield and immunofluorescence micrographs of neurons showed comparable neuronal differentiation ability of WT and *RAP1*$^{-/-}$ hNSCs. Scale bar, 50 µm.

(B) Transwell analysis showed comparable migration ability of WT and *RAP1*$^{-/-}$ hNSCs. Scale bar, 50 µm. Data were presented as mean ± SEM, n = 6. NS, not significant.

Figure S1

A

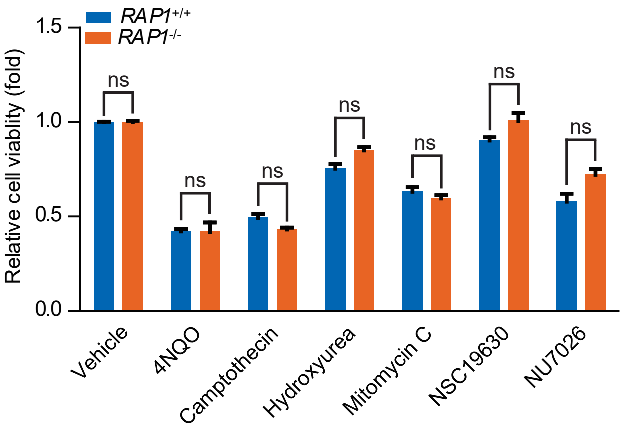

B

**Stressor**	**Main Effects**	**Dose**
4NQO	Inducing DNA damage by producing reactive oxygen species	50 μM
Camptothecin	Inhibiting of topoisomerase and inducing DNA damage by preventing DNA re-ligation	5 μM
Hydroxyurea	Inhibiting DNA replication, inhibiting DNA repair and inducing DNA double strand breaks	50 mM
Mitomycin C	Alkylating DNA and forming DNA cross-links; inhibiting DNA synthesis	100 μM
NSC19630	Inhibiting WRN (a DNA helicase related to DNA repair, replication, telomere maintenance...)	5 μM
NU7026	Inhibiting DNA-PK (a complex related to double-strand DNA breaks repair by NHEJ)	100 mM

Figure S2

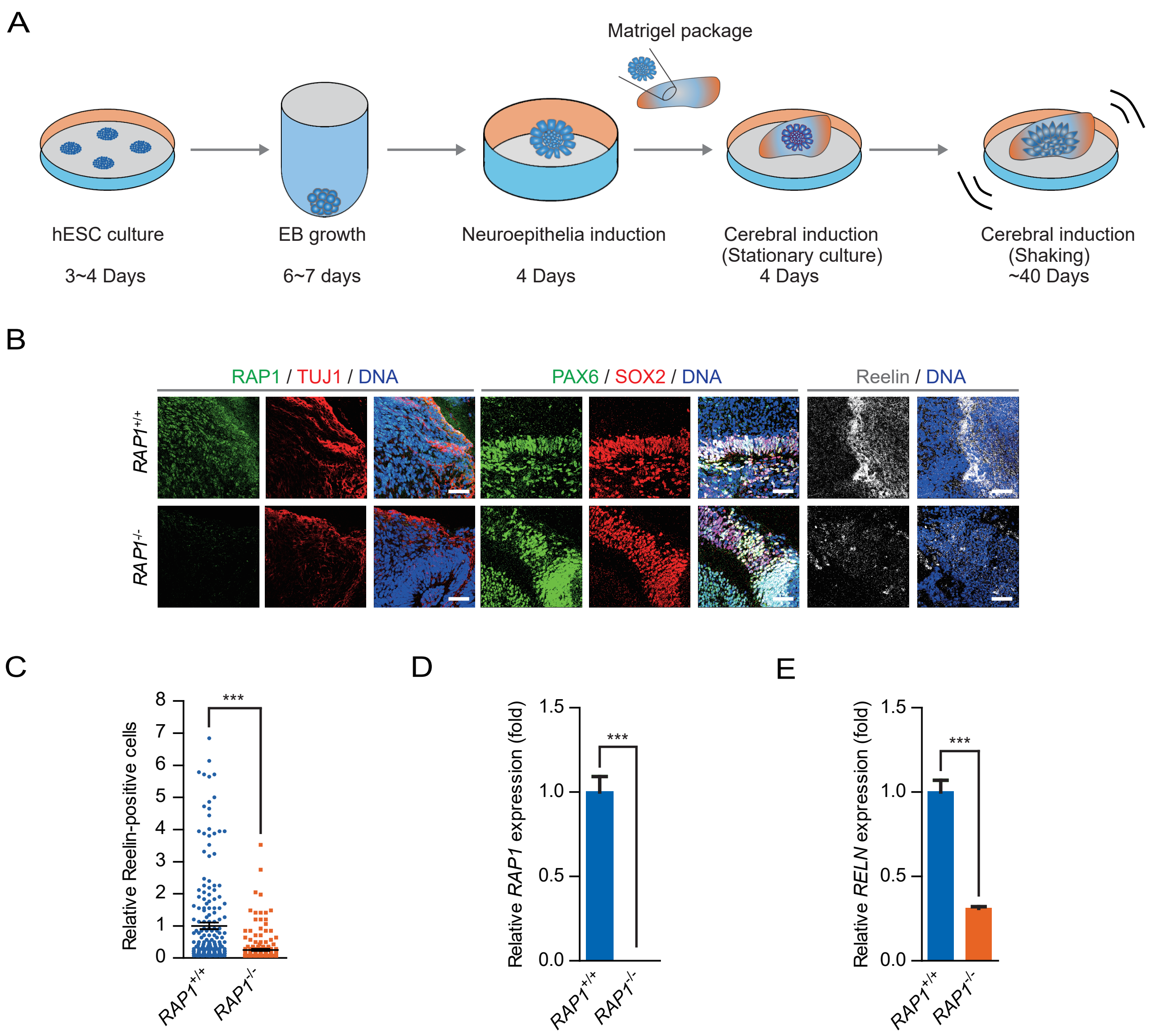

A
Matrigel package
hESC culture
3~4 Days
EB growth
6~7 days
Neuroepithelia induction
4 Days
Cerebral induction
(Stationary culture)
4 Days
Cerebral induction
(Shaking)
~40 Days
B
RAP1 / TUJ1 / DNA
PAX6 / SOX2 / DNA
Reelin / DNA
RAP1+/+
RAP1-/-
C
Relative Reelin-positive cells
***
RAP1+/+
RAP1-/-
D
Relative RAP1 expression (fold)
***
RAP1+/+
RAP1-/-
E
Relative RELN expression (fold)
***
RAP1+/+
RAP1-/-

Figure S3

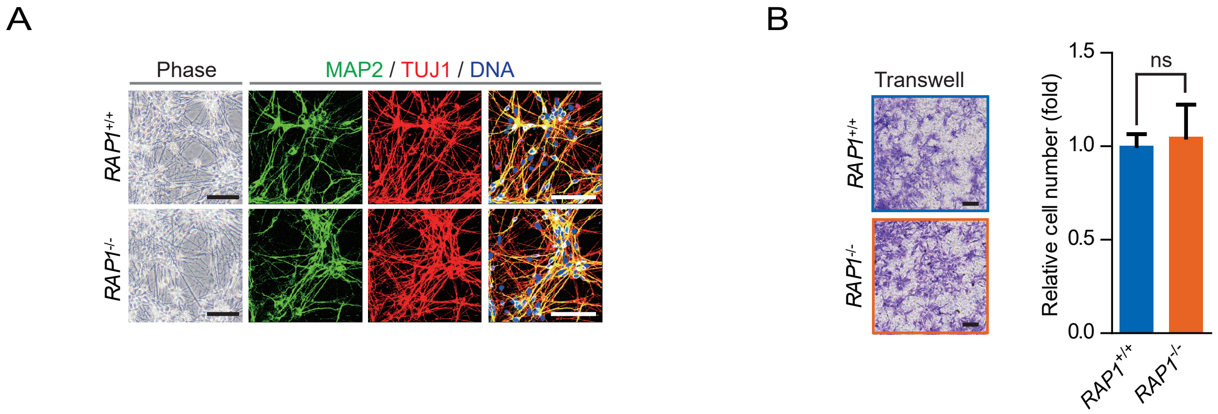
A
Phase
MAP2 / TUJ1 / DNA
RAP1+/+
RAP1-/-
B
Transwell
RAP1+/+
RAP1-/-
1.5
1.0
0.5
0.0
ns
Relative cell number (fold)
RAP1+/+
RAP1-/-
